# Association of Equine Squamous and Glandular Gastric Disease with Dental Status in 54 Horses

**DOI:** 10.3390/ani14223189

**Published:** 2024-11-07

**Authors:** Rabea Lensing, Caroline Wirth, Franziska Thünker, Roswitha Merle, Ann Kristin Barton

**Affiliations:** 1 Equine Clinic Hochmoor, 48712 Gescher, Germany; 2 School of Veterinary Medicine, Institute of Veterinary Epidemiology and Biostatistics, Freie Universitaet Berlin, 14163 Berlin, Germany

**Keywords:** equine gastric ulcer syndrome, omeprazole, dental disorders, horse, pH

## Abstract

Gastric pH is a key factor in the development of equine squamous gastric disease (ESGD). Although acid injury is unlikely the primary cause of equine glandular gastric disease (EGGD), low pH may promote mucosal damage. Gastric pH is influenced by roughage uptake, as chewing increases the alkaline saliva production. The proton pump inhibitor omeprazole is currently recommended by the American/European College of Veterinary/Equine Internal Medicine (ACVIM/ECEIM) for ESGD and EGGD. We hypothesized that dental disorders decrease saliva production and gastric pH, predisposing horses to EGUS. Gastroscopy, intragastric pH measurement and dental examinations were performed (*n* = 54). Omeprazole therapy was recommended (*n* = 32), dental disorders were corrected (*n* = 22) and gastroscopy was repeated 4 weeks later (*n* = 9). An influence of moderate to severe dental disorders on ESGD (grade ≥ 2/4) (*p* = 0.394) and EGGD (grade ≥ 2/3) (*p* = 0.857) could not be determined. Nevertheless, there was a trend that moderate to severe dental disorders were associated with a low gastric pH ≤ 4 (*p* = 0.100). Horses with no or mild dental problems had a mean pH of 5.4 versus 4.2 in moderate to severe dental disorders. In conclusion, our study results do not show a direct relationship between dental disorders and EGUS, but a gastric and oral cavity examination should still be considered in patients presented for weight loss or inappetence.

## Simple Summary

High acidity (low pH) within the stomach has been shown to be important for the development of stomach ulcers in horses, known as equine gastric ulcer syndrome (EGUS). Although acid injury is not thought to be the primary cause of mucosal ulcers, a low pH may perpetuate mucosal damage, causing clinical signs and inhibiting mucosal healing. It is influenced by roughage uptake such as hay and straw. Stomach ulcers are routinely treated with omeprazole, a medication that decreases stomach acid production. In 54 horses, an endoscopic examination of the stomach with measurement of the gastric fluid’s acidity (pH) and an examination of the oral cavity were carried out. In total, 13/21 (61.9%) horses with a low gastric pH (1–4) had moderate to severe dental disorders. Nevertheless, there was no statistical association between dental disorders and EGUS severity.

## 1. Introduction

Gastric ulcers in horses (equine gastric ulcer syndrome, EGUS) is divided into squamous mucosal disease (equine squamous gastric disease, ESGD) and glandular mucosal disease (equine glandular gastric disease, EGGD) [1].

Gastroscopy is the only method to definitively identify gastric ulcers antemortem [2]. Scoring systems have been developed to evaluate changes in the gastric mucosa. According to international consensus, a system from 0 (intact epithelium and no hyperkeratosis) to 4 (extensive lesions with deep ulcerating areas) exists for the cutaneous mucosa [1].

Even though the Consensus Statement [1] deliberately refrained from using a scoring system for EGGD, as visual findings correlate significantly less with clinical signs and prognosis, scoring systems can be useful in the context of scientific studies and for assessing the course of treatment. Scoring systems with various subgrades were therefore also designed to assess the glandular mucosa [3,4,5,6]. EGGD and ESGD can occur simultaneously, but there is currently no clear association between the presence of the two diseases [2].

Pathophysiologically, ESGD is caused by management factors that increase the acid exposure of the squamous mucosa. The sensitivity of the squamous mucosa to hydrochloric acid and volatile fatty acids is pH^−^, time- and dose-dependent. After the initial damage caused by the acid, diffusion into the stratum spinosum leads to ulceration. By-products of bacterial fermentation of carbohydrates exacerbate the damage caused by hydrochloric acid. Dietary fiber is likely to have a dual role in the pathogenesis of ESGD. It increases the saliva production during chewing, which has a buffering effect on gastric acid, and it allows a “forage mat” to form in the stomach, which limits the distribution of acid [7].

In contrast to the squamous mucosa, the glandular mucosa has a number of protective mechanisms, as it is constantly exposed to hydrochloric acid [8]. The pH in the ventral stomach of adult horses is around 2.9 due to the constant secretion of acid. In contrast, the pH in the dorsal stomach is less acidic (mean pH: 6.8) and subject to strong fluctuations, presumably due to feeding behavior. During the day, horses have a significantly higher pH in the dorsal stomach [2]. The pathophysiology of glandular gastritis is not yet sufficiently understood. Possible factors contributing to EGGD are the breakdown of mucosal defenses, bacterial colonization, stress and inflammation [8], resulting in the loss of the protection of the mucosa against acid exposure. Although acid injury is not thought to be the main cause of EGGD, a low pH can initiate mucosal damage and inhibit mucosal healing [9].

Oral omeprazole (given in a buffered or enteric-coated form) is licensed for the treatment of gastric ulceration in many countries and is an effective agent for the management of ESGD [10,11]. For the treatment of EGGD, a combination therapy of omeprazole at a dosage of 4 mg/kg PO SID and sucralfate at a dosage of 12 mg/kg PO BID is a valid first-line treatment [9], although scientific evidence regarding its efficacy is lacking in the literature [12].

The prevalence of ESGD varies greatly depending on exercise, training condition, breed, sex and age, and can range from 11% to 100% [13]. The prevalence of EGGD varies between 6% and 72%, depending on the studied population [7].

The literature shows that slow and prolonged feed intake leads to a continuous flow of saliva and thus to a better buffering capacity in the stomach [14]. If horses are starved, the pH in the stomach decreases [15]. Previous studies have shown that feed deprivation (alternating 24 h intervals of fasting and feeding) resulted in ESGD [16,17,18].

Dental problems are the third or fourth most common reason owners present their horses for veterinary treatment in the USA [19]. Dental lesions are thought to lead to inefficient mastication, resulting in reduced feed conversion and weight loss, an increased risk of intraluminal esophageal obstruction and intestinal obstruction, and occasionally diarrhea [20].

Some studies have shown high levels of clinically significant, non-diagnosed dental disorders in horses [21,22]. Due to oral pain, some horses may also chew very slowly and may be reluctant to eat hay or silage (haylage). In extreme cases, feed intake is reduced and therefore possibly also saliva production. The most common equine dental (and oral) disease is the development of sharp dental overgrowths and may cause lacerations of the cheeks and tongue during chewing [23].

Floating (rasping or filing) of the teeth is still the most frequently performed dental procedure [20]. Removing mechanical impediments to lateral chewing movements and painful dental overgrowth promotes normal chewing movements and helps restore normal oral food and saliva flow. The aim of routine dental care should be to prevent the development of such end-stage disease by regular dental inspections every 6–12 months [23].

Despite the many studies that have been published on the subject of gastric ulcers in horses, none have so far investigated a possible association with disorders of the oral cavity. In this observational study, we investigated whether moderate to severe disorders in the oral cavity led to a lower gastric pH (≤4) and gastric ulcers in horses.

## 2. Materials and Methods

### 2.1. Animals

This prospective clinical study included 54 horses of various ages (from 2 to 28 years), sexes (mares: 27; stallions: 1; geldings: 26) and breeds, which were presented for gastroscopy at the Equine Clinic Hochmoor, Germany, and had an oral cavity examination under the same sedation between September 2023 and April 2024. The horses had a history of recurrent colic, weight loss, loss of appetite, fecal water, exercise insufficiency or typical gastric discomfort behavior (bruxism, groaning and hypersensitivity/aggressive behavior in response to grooming, the rider’s leg or fastening the girth). Horses were starved overnight for approximately 12 h for feed and 3 h for water withdrawal. Most of the patients were stabled in the clinic the day before the examination and were given standardized hay or were already in the clinic due to colic therapy. Some horses were presented as ambulatory patients and fasted at home. Nine of the horses with obvious findings in the stomach were presented again for a gastroscopic follow-up after treatment with omeprazole and two of them with additional dental treatment.

### 2.2. Gastroscopic Examination

The horses were sedated intravenously with 0.012 mg/kg bw detomidine (Domidine™ 10 mg/mL, Dechra Veterinary Products Deutschland GmbH, Aulendorf, Germany) and 0.025 mg/kg bw butorphanol 0.025 mg/kg (Torbugesic™ VET 10 mg/mL, Zoetis Deutschland GmbH, Berlin, Germany). For the gastroscopy, a flexible endoscope of 330 cm length and 1.3 cm diameter (Karl Storz GmbH, Tuttlingen, Germany) was used, and the stomach was insufflated with air for complete visualization of the relevant structures (margo plicatus, small curvature and pylorus). During gastroscopy, a few milliliters of the gastric fluid in the ventral stomach were aspirated through the working channel and the pH value was measured using pH test strips (pH indicator strips, MColorpHast^TM^, Merck, Darmstadt, Germany). Food material adhering to the stomach was flushed away with water after measurement of the pH. The gastric findings were recorded by two veterinarians (a diplomate and a resident ECEIM) at time of examination and divided into grades using scoring systems (Table 1 and Table 2).

### 2.3. Oral Cavity Examination

Following gastroscopy, an oral examination was carried out by a veterinary surgeon under the same sedation. The findings were documented and categorized into 4 grades depending on the degree of severity, the number of abnormalities and influence on the occlusion (Table 3 and Supplementary Materials Table S1). A low degree of severity includes anomalies that have little or no negative influence on occlusion, such as a cribbing bite. Severe dental abnormalities, such as multiple missing teeth, can affect the occlusion negatively. Hooks and edges can impede the normal movement of the jaw and were therefore graded as mild, moderate or severe depending on their severity.

### 2.4. Owner Questionnaire

The owners were given a questionnaire (the original questionnaire was prepared in German and is available in English in the Supplementary Materials Figure S1). Questions included general information about the horse, stable management, feeding and use, as well as its medical history, behavioral problems and stress factors, dental treatment status, gastric health and any previous treatments.

In order to analyze the influence of confounders, particularly previous gastric treatment with omeprazole and previous dental treatments, these variables were recorded in the questionnaire. The aim of these surveys was to identify possible distortions in the results and to analyze their effects on the horses’ gastric pH.

### 2.5. Treatment

For horses presenting with ESGD grade ≥ 2/4 and/or EGGD grade ≥ 2/3, omeprazole treatment was commenced according to the recommendations of the Consensus Statement (buffered formulation, Gastrogard™, Boehringer Ingelheim Germany, Ingelheim, Germany; 4 mg/kg PO SID or 2 mg/kg enteric coated granules, Equizol™, CP Pharma Germany, PO SID, Burgdorf, Germany, both over 4 weeks). An overview of the gastric/dental findings and recommended treatment for each horse is available in the Supplementary Materials Table S1. In addition, the teeth were floated and diastemata were cleaned where indicated. The owners were given management recommendations, such as avoiding long periods of food withdrawal and feeding hard feed after roughage. In order to monitor the success of the treatment, follow-up gastroscopy was recommended at the end of the therapy (about 4 weeks later).

### 2.6. Statistical Analysis

Clinical data were recorded in a digital patient documentation system (easyVET™, VetZ Gmbh, Isernhagen, Germany) and Microsoft Excel™. The IBM SPSS Statistics 29.0.1.0 program was used for the descriptive evaluation of the data, as well as statistical analysis and chart creation. A value of *p* < 0.05 was considered significant. The ESGD (≤1/4 and ≥2/4), EGGD (≤1/3 and ≥2/3) and oral cavity score (≤1/3 and ≥2/3), as well as the gastric pH value (≤4 and ≥5), were combined into larger supergroups in order to increase the number of cases per group. Initially, descriptive statistics were carried out, including the creation of a cross-table (Table 4). Continuous data were assessed for normality, and Chi square tests were used to analyze the possible influences of the findings in the oral cavity (no to low, or medium to high findings) on the ESGD, EGGD and gastric pH supergroups, respectively (Table 4). Logistic regression was applied to analyze the association between the binary variables in more detail. In addition, the confounding variables of pretreatment of the stomach with omeprazole within the week prior to gastroscopy and time since last dental treatment (categorized as <6 months, 6–12 months and >12 months) were investigated. Patients with missing data were excluded for the individual analyses and patient-related factors were not included.

## 3. Results

At the first examination, 55.6% (*n* = 30/54) of the horses had ESGD grade ≥ 2/4, the stomach was fully visible with EGGD grade ≥ 2/3 in 17.0% (9/53), and 13.2% (7/53) had both ESGD and EGGD with scores ≥ 2. The exact distribution is shown in Table 5. Therapy with omeprazole (Gastrogard™ or Equizol™) was recommended in 59.3% (32/54) of patients (Supplementary Materials Table S1). Gastrogard™ was administered to 21 horses and Equizol™ to 11 horses.

Moderate or severe findings in the oral cavity were initially seen in 48.1% (26/54). Of these, 19 and additionally the teeth of 3 horses with low-grade findings were treated (table with individual findings in the Supplementary Materials Table S1). The gastric pH ranged between 1 and 9, and 40.4% (21/52) of the horses had gastric pH ≤ 4, and 59.6% (31/52) had pH ≥ 5 (Table 4). Gastric pH was not measured in 2/54 horses, as they had gastric lavage prior to gastroscopy. ESGD scoring was performed in all horses (*n* = 54); in one horse, EGGD scoring was not possible, as the glandular mucosa was not sufficiently visible (*n* = 53).

Thirty-four out of fifty-four (63%) patient questionnaires were answered and used to assess the confounding variables. The questionnaire revealed that most horses were kept in boxes (19/34), with 16 horses spending several hours a day at pasture. Most horses were pleasure (22/34) and 10/34 active sports horses. Hay, haylage or silage were fed two to three times a day (20/34) to most horses and they received hard feed at feeding times. The majority of patients were dewormed two to three times a year (24/34), and three were dewormed selectively. Many owners (21/34) stated that there had been stressful situations for the horse recently, e.g., rank fights during herd changes or competitions, or that the horse was generally nervous. The remaining owners were not aware of any stressful situations (an overview table of the questionnaire can be found in the Supplementary Materials Table S2). For 48 horses, it was known whether they had been treated with omeprazole in the previous week, of which 6 had received omeprazole. Information on the last dental treatment could be provided by questionnaire or directly from the owners of 38 patients. Among these, 21 horses had received treatment within the last 6 months, 12 horses between 6 and 12 months and 5 horses more than 12 months prior.

**Figure 1:**
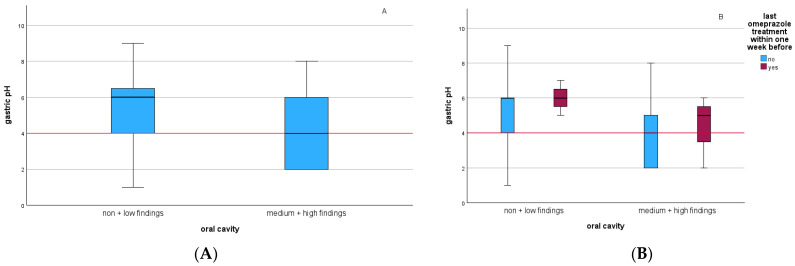
Oral cavity findings in relation to gastric pH of all study participants. The red marking indicates the division into the two groups for the statistical analysis. (**A**,**B**) Subdivision of the oral cavity findings in horses which the owners stated had been pretreated with omeprazole within the week prior to this study (red) or had not been pretreated (blue) (**B**).

The univariable logistic regression indicated that moderate to severe dental disorders decrease gastric pH ≤ 4, although not statistically significantly (*p* = 0.104; odds ratio (OR) = 2.571; 95% confidence interval (CI) 0.824–8.065) (Table 5). Including the confounder omeprazole pretreatment, the odds ratio increased (OR = 4.717; 95% CI 1.292–17.241) and it became significant (*p* = 0.019) (Figure 1 and Supplementary Materials Table S2). There was no statistically significant association between dental disorders (moderate to severe) and ESGD (≥2/4) (*p* = 0.395; OR = 1.600, 95% Cl 0.542–4.726) (Table 5). Including the confounders, the odds ratio increased and the *p*-value decreased, but there was still no significance (last dental treatment: OR = 2.513; 95% CI 0.552–11.432; *p* = 0.233; omeprazole pretreatment: OR = 2.671; CI 0.748–9.534; *p* = 0.130). No association was found for dental disorders (moderate to severe) and EGGD (≥2/3) (*p* = 0.857) (Table 5). Furthermore, no association between the omeprazole pretreatment and ESGD (≥2/4) (*p* = 0.334) or EGGD (≥2/3) (*p* = 0.581) could be demonstrated.

Nine patients were presented for a gastroscopic follow-up after approximately 4 weeks. They were treated with omeprazole, and two of them also had their dental disorders corrected. ESGD was improved by at least two subgrades or achieved grade 0 in 5/9 horses. Of patients with EGGD, 3 out of 8 fully visible stomachs showed a reduction of at least one subgrade (Table 6).

## 4. Discussion

To our knowledge, this study is the first to investigate a possible relation between dental and gastric disease.

The majority of horses in our study, which were examined due to a clinical suspicion of EGUS, showed at least grade 1/4 ESGD and at least grade 1/3 EGGD. Depending on breed and performance, a prevalence of EGUS of 37–100% for ESGD and from 6% to 72% for EGGD has been described [7]. Due to this wide range, our results of 83% for ESGD and 59% for EGGD are consistent with this. As the horses in our study were examined after presenting typical clinical signs, this high prevalence is to be expected, although a former study in horses with a history of comparable clinical signs that also presented for gastroscopy at a clinic showed a lower ESGD and higher EGGD case rate [4].

A possible explanation might be that our study period from September to April excluded the summer season, which can lead to gastric ulcers in competition horses due to increased training and participation in competitions [24]. On the other hand, most horses spend longer periods on pasture during the summer months, which can potentially lead to shorter feeding pauses.

In our study, ESGD was graded from 0 to 4 as previously recommended [1,3]. EGGD was graded from 0 to 3, as modified from the Consensus Statement [1] and already used by Barton et al. [4]. The oral cavity score was developed on the basis of the findings, and the potential influence on function as described in the literature [20,23,25,26]. It should be mentioned that the veterinarian performing the dental examination was not blinded to the gastric ulcer diagnosis. In order to obtain a larger number of horses per group and therefore to increase the statistical power, the ESGD, EGGD and oral cavity scores, as well as the fasted gastric pH, were combined into two supergroups, and the statistical calculations were carried out with these. Classification of data leads to loss of information but might be necessary to detect effects at all. We decided to classify ESGD, EGGD, oral cavity score and the gastric pH below and above threshold because there were not so many observations and because we wanted to analyze if elevated scores had an effect at all. The division of the ESGD score into ≤1/4 and ≥2/4 was chosen because a score of 2/4 or higher can be considered clinically significant [27]. In the case of EGGD, studies reported spontaneous healing of low grades [28], which is why the grades 0–1/3 and grades 2–3/3 framework was chosen. The classification of the gastric pH into 1–4 and 5–9 was chosen, as in various other studies, the limit was also set at >4 [24,29]. Furthermore, ESGD and/or EGGD ulcers have been shown to heal faster with an intragastric pH of >4 [27]. The chosen group categorization of the oral cavity resulted in a homogeneous distribution and divided the horses into those with unlikely influence (grades 0–1/3) and those with a likely influence on the chewing process (grades 2–3/3). We also assumed an impairment in cases of multiple low-grade findings. However, as the categorization was subjectively determined and moderate interobserver agreement may have occurred, as formerly shown [4], this effect remained insignificant.

ESGD treatment was recommended to start at grade 2, as hyperkeratosis (grade 1) in the horse’s stomach may be considered as a “normal” reaction to acid exposure and as ESGD has also been found in feral horses that are not exposed to any recognized risk factors [4,28]. In addition, many horses show no clinical signs despite ESGD findings, and mild lesions can heal spontaneously [30,31].

Gastrogard™ is a buffered formulation which, according to the manufacturer’s instructions, is to be administered at a dosage of 4 mg/kg, while Equizol™ is available as enteric-coated granules and is recommended by the manufacturer at a dosage of 2 mg/kg due to its increased bioavailability [4].

There are variable study results on the healing and improvement rates of ESGD and EGGD with 4 weeks of omeprazole therapy, although the trend is the same. ESGD shows a better healing tendency than EGGD. We included horses treated with Equizol™ at 2 mg/kg and Gastrogard™ at 4 mg/kg, as former studies have shown no difference [4,12]. The Consensus Statement reports cure rates for Gastrogard™ of 78% for ESGD and 25% for EGGD. More recent studies [32,33,34] show, depending on the definition used, cure rates for omeprazole therapy of between 59% and 67% for ESGD and between 25% and 50% for EGGD. The patients who underwent gastroscopic follow-up in our study showed comparable cure rates, with 55.6% for ESGD and 37.5% for EGGD. The influence of dental treatment on the patients who were presented for repeat gastroscopy cannot be assessed due to the small number of cases.

According to Jenkins et al. [35], gastric acid secretion is significantly inhibited by omeprazole for 27 h. In our study, horses with poor oral cavity health and no recent omeprazole treatment (within one week prior to gastroscopy) were found to have a significantly lower gastric pH. However, it is important to note that only a small number of horses (*n* = 6) had received omeprazole pretreatment, which limits the statistical power. Although oral cavity health showed a trend to have an influence on stomach pH, a relationship between oral cavity health and ESGD could not be statistically demonstrated. Similarly, omeprazole pretreatment had no significant effect on the correlation between oral cavity health and ESGD. No association was seen between the oral cavity health and the EGGD score and between the omeprazole pretreatment and ESGD or EGGD. Despite statistical evidence suggesting that the administration of omeprazole in the week prior to gastroscopy, combined with good oral cavity health, increases gastric pH, a possible alternative explanation is that the observed significant results could be attributed to the implementation of a gastric-optimized stable management and feeding regime, rather than the administration of the relatively short-acting omeprazole. Although the gastric pH increased, the omeprazole pretreatment did not lead to a significant improvement in the EGUS score. This may be attributed to the fact that the ulcers did not have sufficient time to undergo the healing process, the scoring system did not offer enough detail for differentiation within the grade, or they had subepithelial and non-visible healing processes.

A previous study showed that severe ESGD can be improved or even treated successfully within 4 weeks without drug therapy by providing horses with roughage ad libitum and a small amount of a low-starch supplement. A predictable daily routine with a limited number of good caretakers can contribute to lower stress levels and thus improve gastric health [36]. Another study has shown that periods of feeding breaks over 6 h increase the risk of ESGD [37], as well as high starch intakes, because starch leads to an increased production of volatile fatty acids (VFAs), which reduce the integrity of the mucous membrane [38].

Additionally, the teeth of 22 horses (19 horses with moderate to severe findings and 3 with mild findings at the owners’ request) were treated. No treatment was carried out on the remaining patients despite moderate to severe dental findings. Two of these horses had too smooth chewing surfaces, and one horse had missing teeth without additional treatable findings. Some owners decided to have the already scheduled dental treatments carried out by their own equine dentist.

In our study, the mean pH in stomach fluid was 4.81, which was higher than in other studies [39]. Especially when horses are fasted, the pH should be lower; in one study, it was 1.55 in fasting compared with 3.1 in fed horses [15]. A possible reason for the high pH values measured in this study could be contamination with water, the use of insensitive pH strips or previous treatment with omeprazole. It is also possible that our pH samples were collected higher in the gastric contents where the pH is less acid. In addition, we chose a short fasting period to identify potential indications of a possible gastric emptying disorder. Nevertheless, almost all stomachs were empty, indicating that the pH may not have decreased sufficiently due to the relatively short fasting period. Some patients were starved at home, where implementation was not monitored, but a well-emptied stomach could be considered as successfully fasted at gastroscopy.

The questionnaire was designed in a similar way to comparable studies [13] and was returned by >60% of owners. Some questions were answered incompletely, sometimes because the horse had not been owned for long or because it was not known better. The reliability of other statements should also be treated with caution, as they were not checked further. For example, the indication of the last dental treatment, which was divided into the usual intervals [23], should not be given too much weight, as it was not absolutely clear that dental treatment had been carried out or that only an examination was performed.

It should be noted that some categories had incomplete survey data due to the reasons mentioned above. Patients with incomplete data were excluded from the individual statistical analysis, which reduced the sample size and thus the statistical power in some cases.

This study suffered from several limitations. Due to its nature of being an observational study with horses presented to the clinic, only 54 horses were included in this study, and there was no blinding or proper randomization of the treatment possible. Also, the survey data were not always complete, and fasting of the horses in their home stable could not be controlled. Thus, statistically significant or almost significant effects need to be interpreted with care. But this study’s results indicate that further investigations in a more controlled approach might be of value.

## 5. Conclusions

In conclusion, this study showed no influence of moderate to severe dental disorders on ESGD (≥2/4) and EGGD (≥2/3). Although moderate to severe dental disorders tended to be associated with a low gastric pH (≤4), this was not statistically significant. It was statistically significant that horses without dental problems or low-grade findings which had previously been given omeprazole within one week prior to the investigation had a higher gastric pH. However, this effect is unlikely to be due to the short-acting omeprazole. Although our study results do not show a direct relationship between dental disorders and EGUS, a gastric and oral cavity examination should be considered in patients with signs of weight loss or inappetence. Further studies with a larger number of cases and a longer study period are required.

## Figures and Tables

**Table 1:** Grading system for equine squamous gastric disease (ESGD) from the ECEIM Consensus Statement (Sykes et al. 2015, adapted from Andrews et al. 1999a) [1,3]. The dotted line indicates further division into supergroups.

Grade	Squamous Mucosa
0	Intact epithelium and no appearance of hyperkeratosis
1	Intact mucosa, but areas of hyperkeratosis
2	Small, single or multifocal lesions
3	Large single or extensive superficial lesions
4	Extensive lesions with areas of apparent deep ulceration

**Table 2:** Grading system for equine glandular gastric disease (EGGD), modified from the ECEIM Consensus Statement (Sykes et al. 2015) [1] and Barton et al. 2022 [4]. The dotted line indicates further division into supergroups.

Grade	Glandular Mucosa
0	Intact mucosa
1	Intact mucosa, patchy or streaky yellowish or reddish lesions
2	Small, isolated or multifocal lesions
3	Large single or extensive superficial lesions, possibly bleeding

**Table 3:** Grading system for oral cavity health. The dotted line indicates further division into supergroups. A score of grade ≥ 2/3 is labelled as moderate to severe dental/oral cavity disorders.

Grade	Oral Cavity
0 (no findings)	No special findings
1 (low findings)	≤2 low-grade abnormalities
2 (medium findings)	Medium-grade abnormalities or ≤4 low-grade abnormalities
3 (high findings)	High-grade abnormalities or >4 low-grade abnormalities

**Table 4:** Relationship between oral cavity findings (non-specific to low or moderate to severe) and gastric pH, ESGD and EGGD scores, with significance value determined by Chi² test.

	Non-Specific to Mild Dental Disorders	Moderate to Severe Dental Disorders	*p*-Value (Chi² Test)
pH 1–4	8/21 (38.1%)	13/21 (61.9%)	0.100
pH 5–9	19/31 (61.3%)	12/31 (38.7%)	
ESGD ≤ 1/4	14/24 (58.3%)	10/24 (41.7%)	0.394
ESGD ≥ 2/4	14/30 (46.7%)	16/30 (53.3%)	
EGGD ≤ 1/3	23/44 (52.3%)	21/44 (47.7%)	0.857
EGGD ≥ 2/3	5/9 (55.6%)	4/9 (44.4%)	

**Table 5:** Frequency of occurrence of the oral cavity and ESGD/EGGD scores and gastric pH. The dotted line indicates further division into supergroups.

Oral Cavity Score	No. Horses (% of 54)	ESGD Score (0–4)	No. Horses (% of 54)	EGGD Score (0–3)	No. Horses (% of 53)	Gastric pH	No. Horses (% of 52)
						1	1 (1.9)
						2	11 (21.2)
0	8 (14.8)	0	9 (16.7)	0	22 (41.5)	3	3 (5.8)
1	20 (37.0)	1	15 (27.8)	1	22 (41.5)	4	6 (11.5)
2	17 (31.5)	2	10 (18.5)	2	8 (15.1)	5	9 (17.3)
3	9 (16.7)	3	16 (29.6)	3	1 (1.9)	6	12 (23.1)
		4	4 (7.4)			7	4 (7.7)
						8	5 (9.6)
						9	1 (1.9)

**Table 6:** ESGD and EGGD scores before (first examination) and after omeprazole treatment (second examination) in the nine horses presented for follow-up examination. In one case, a third gastroscopy was performed after further therapy.

	First Examination		Second (Third) Examination	
Horse ID	ESGD (0–4)	EGGD (0–3)	ESGD (0–4)	EGGD (0–3)
2 *^,g^	4	2	1 **	n.a.
5 ^g^	3	3	2	1 ***
15 ^g^	3	1	0 **	0 ***
25 ^e^	3	1	0 **	1
32 ^g^	4	0	4 (2 **)	0 (0)
34 ^e^	4	1	2 **	1
35 *^,e^	3	1	2	1
39 ^g^	3	1	0 **	1
50 ^e^	3	2	3	1 ***

In two patients, the teeth were treated in addition to the omeprazole therapy (*). The stomachs were treated with Gastrogard™ (^g^) or Equizol™ (^e^); ESGD was improved by ≥2 subgrades or achieved grade 0 in 5/9 horses (**) and EGGD was improved by ≥1 subgrade in 3/8 horses (***). In one patient, the glandular mucosa of the stomach could not be fully visualized in the follow-up examination (n.a.).

## Data Availability

Data are contained within the article or Supplementary Materials; further inquiries can be directed to the corresponding author/s.

## Supplementary Materials

The following supporting information can be downloaded at https://www.mdpi.com/article/10.3390/ani14223189/s1. Figure S1: Questionnaire. Table S1: Table with gastric and dental findings and their scores and therapy. Table S2: Overview table of the questionnaire’s results. Table S3: Regression table.
