# Supplementary material for: Association of Equine Squamous and Glandular Gastric Disease with Dental Status in 54 Horses
Source: Animals (Basel). 2024 Nov 7;14(22):3189. doi: 10.3390/ani14223189 (PMC11591547; doi:10.3390/ani14223189)
Supplement: Supplementary file 1 [file animals-14-03189-s001.zip › FigureS1. Questionnaire 01.11.2024.pdf]

Dear patient owners,

My name is Rabea Lensing and I am a veterinarian at the Equine Clinic Hochmoor.

As part of my doctoral thesis at the Free University of Berlin, I am working on the topic „Influence of oral cavity health on gastroscopic findings".

Your horse will untergo a gastroscopy at the Equine Clinic Hochmoor. I would like to carry out an examination of the oral cavity under the same sedation in order to obtain information for my doctoral thesis. If the examination reveals any findings that require treatment, you will be informed and can be treated immediately if necessary.

If you agree to the oral cavity examination, please complete the following questionnaire:

Name (patient owner): ____

Details of the horse:

Name		
Age		
Sex		
Breed		
Weight		
Size		
Owned since		
Posture	Boxen housing □ Paddock box housing □<br>Grazing □ ______ hours/day Robust husbandry □<br>Open stable housing □ Active stable housing □<br>Bedding material: ________________________________<br>How many other horses?:____________________________<br>Other:____	
Usage	Leisure □ Sport □	
	Tournament participation	yes □ no □
	Training level	
	Training frequency	Hours/week
	Number of riders	
Feeding	What?	
	How much?	
	How often?	
	How? (e.g. hay net, watered hay)	
	In what order?	
	Access to water (e.g. always, limited)	

Pre-treatment<br><br>What medication (stomach medication, painkillers) has your horse been given in the last 6 month?	What?	
	How much?	
	How long?	
	When was the last time?	
Oral cavity examinations/ Dental treatment	How often?	
	When was the last time?	
	What was done?	
Has your horse ever had a gastroscopy?	Yes □<br>With the result: ____<br><br>No □	
Deworming	How often?	
	When was the last time?	
	Which?	
Signs of illness (e.g. drop in performance, flehmen, faeces water, colic symptoms), behavioral abnormalities (e.g. cribbing)	Which?	
	How strong?	
	When? (e.g. after feed intake)	
Have there been any stressful situations for your horse recently (e.g. change of stable, transportation, ranking problems, participation in competitions)?	Yes □, the following situation(s):____<br><br>No □, I am not aware of this.	

Since the success oft he treatment is also of great interest, I would like to contact you after some time to check your horse´s eating behavior and willingness to perform, for example.

I agree to the examination of my horse´s oral cavity and to the anonymized participation in Rabea Lensing´s dissertation project.

___________________________________ ____

Place, date Name, signature

Thank you for your participation!
