# Supplementary material for: Association of Equine Squamous and Glandular Gastric Disease with Dental Status in 54 Horses
Source: Animals (Basel). 2024 Nov 7;14(22):3189. doi: 10.3390/ani14223189 (PMC11591547; doi:10.3390/ani14223189)
Supplement: Supplementary file 1 [file animals-14-03189-s001.zip › Table S3. Regression table supplements 01.11.2024.pdf]

Table S3. Variables in the Equation

		B	S.E.	Sig.	Exp(B)	95% C.I.for EXP(B)	
						Lower	Upper
Step 1[a]	oralcavity_group(1)	-1.553	.662	.019	.212	.058	.774
	last omeprazole treatment within one week before(1)	1.617	1.195	.176	5.038	.484	52.399
	Constant	.966	.480	.044	2.627		

a. Variable(s) entered on step 1: oral cavity group, last omeprazole treatment within one week before.
